# The effect of aging and muscle type on the quality characteristics and lipid oxidation of lamb meat

**DOI:** 10.5194/aab-62-383-2019

**Published:** 2019-07-04

**Authors:** Witold Rant, Aurelia Radzik-Rant, Marcin Świątek, Roman Niżnikowski, Żaneta Szymańska, Magdalena Bednarczyk, Emil Orłowski, Anna Morales-Villavicencio, Magdalena Ślęzak

**Affiliations:** Department of Animal Breeding and Production, Warsaw University of Life Sciences-SGGW, Ciszewskiego 8, 02-786 Warsaw, Poland

## Abstract

The research carried out on meat from 45 ram lambs of the Polish merino breed allowed to determine the effect of meat aging and muscle type on physicochemical characteristics and oxidative stability of lipids. Analysis of physicochemical traits (pH, meat color, expressed juice, cooking loss, shear force, moisture, protein, fat and total collagen content) was performed on fresh and meat aged for 14 d in the *longissimus lumborum* (LL) and *gluteus medius* (GM) muscles. The meat aging determined all physicochemical characteristics except protein and fat content. More changes in pH and meat color parameters were defined in the GM muscle compared to the LL muscle. The increase in the tenderness of meat expressed as a reduction (P<0.05) of shear force values was observed in both muscles aged for 14 d. A lower value (P<0.05) of the shear force, despite the higher content of collagen, was determined in the GM muscle compared to LL. The investigated muscles differed in the degree of lipid peroxidation expressed as thiobarbituric acid-reactive substances (TBARS) in both fresh and aged meat. The TBARS value was lower (P<0.05) in the LL muscle than in GM. In the *longissimus lumborum* muscle, the significantly lower content of polyunsaturated fatty acids (PUFAs) and PUFA n-6 has been recorded. The oxidation stability was not influenced by the meat aging.

## 1 Introduction

The main factor that determines the consumer acceptance of meat is its quality, which is perceived by such physicochemical features as color, tenderness, juiciness or the amount of intramuscular fat. These properties depend on the animal species and breed, feeding system and body weight at slaughter, as well as muscle type and also on meat aging time (Martinez-Cerezo et al., 2005; Abdullah and Qudsieh, 2009; Callejas-Cardenas et al., 2014; Ablikim et al., 2016; Ponnampalam et al., 2017; Sosin-Bzducha and Puchała, 2017; Florek et al., 2018).

Mammalian muscles are composed of fibers of different morphological, metabolic and functional characteristics (Sazili et al., 2005). The development of various fiber types of muscle affects not only the overall muscle mass in carcasses of slaughter animals but also the quality traits of meat (Lee et al., 2010). The diversity of muscles, due to various proportions of each fiber type in them, may be related to intramuscular fat and connective tissue content, and also has an impact on meat color and water-holding capacity (Picard et al., 2002; Purslow, 2005; Yancey et al., 2005; Ryu and Kim, 2006).

Apart from the features influencing the technological value of meat, the muscle type may contribute to its health value, which includes the content of polyunsaturated fatty acids (PUFAs) (Janovska et al., 2010). The quality of meat for consumption is inseparably linked with the process of its aging after slaughter. During aging, a number of changes occur in the micro- and ultra-structure of the meat which determine the tenderization initiated by the calcium-dependent calpain system (Koohmaraie and Geesink, 2006). The process of meat aging is also associated with favorable changes in its water-holding capacity (Farouk et al., 2012). Apart from the undeniably beneficial effects of post-slaughter aging of meat, there may be processes negatively affecting its final value, resulting mainly from oxidation of lipid components as well as destabilization of meat color (Bekhit et al., 2013; Hopkins et al., 2013; Ponnampalam et al., 2017).

The aging time of meat, which determines the quality indicators, varies depending on the animal species, the cooling conditions and the type of muscle. The expected aging time for beef is determined as 6 weeks (Sosin-Bzducha and Puchała, 2017), whereas according to Nowak (2005) the appropriate aging time for pork is 6–10 d and for poultry meat from 12 to 24 h. The effect of aging time on physicochemical properties of lamb meat was studied in different periods, starting from 7 to even 30 and 60 d after slaughter (Abdullah and Qudsieh, 2009; Ablikim et al., 2016; Ponnampalam et al., 2017).

The aim of this study was to analyze the physicochemical characteristics and lipid oxidation of fresh and 14 d post-slaughter aged lamb meat, taking into account the type of muscle.

## 2 Material and methods

This research was conducted on 45 ram lambs of Polish merino breed fattened to achieve their slaughter weight of 40 kg (±1.5 kg).

The ethical approval for this experiment was obtained from the II Local Ethical Commission for Animal Experimentation in Warsaw (consent form number WAW2_20/2016). The animals were kept in a barn on straw bedding under uniform environmental conditions with constant zootechnical and veterinary supervision.

The lambs were fed in a group according to the standards for fattening lambs up to 30–40 kg (Osikowski et al., 1998). They received the meadow hay, steamed potatoes and concentrate containing 57.8 % oatmeal, 17.6 % wheat bran, 23,5 % rapeseed meal and 1 % mineral mixture. The chemical composition and nutritional value of the fodder are presented in Table 1. The animals were fed twice a day at 07:00 and 16:00 LT (local time). The meadow hay was fed to the lambs separately. The potatoes and concentrate were mixed before each feeding. The animals had constant access to water.

**Table 1:** The chemical composition and nutritional value of feeds used in lamb fattening.

Specification	Grass	Oatmeal	Rapeseed	Wheat	Steamed
	hay		meal	bran	potatoes
Dry matter (g kg${}^{-1}$)	870.4	88.8	902.1	887.3	192.8
Crude protein (g kg${}^{-1}$ DM)	129.8	97.7	325.5	134.6	115.0
Ether extract (g kg${}^{-1}$ DM)	22.0	31.8	34.8	31.6	3.0
Crude fiber (g kg${}^{-1}$ DM)	317.0	90.5	104.6	62.8	37.0
Ash (g kg${}^{-1}$ DM)	36.5	22.6	67.6	43.7	52.9
EN (MJ/1 kg DM)	3.72	6.56	7.47	5.87	8.87

EN: net energy; MJ: megajoule; DM: dry matter.

After reaching the desired body weight lambs were taken to an abattoir and slaughtered according to Council Regulation (EC) no. 1099/2009 of 24 September 2009 (Acts. Office EU dated. 18.11.2009 L 303/1). Carcasses were suspended from the Achilles tendon and chilled at 4 ${}^{\circ }$C for 24 h and weighed. From the right and left sides of each carcass, the samples of *longissimus lumborum* (LL; n=90) and *gluteus medius* (GM; n=90) muscle were collected and vacuum packed. The 45 LL and 45 GM samples were transported in the refrigerator to the laboratory in order to perform quality analysis on not aged (fresh) meat. The other 45 LL and 45 GM muscles were aged in the constant temperature of 2 ${}^{\circ }$C and 68 % humidity for 14 d. After that period, samples were subjected to analysis of meat quality.

### 2.1 Sample preparation and analysis

The analyzed LL and GM samples were trimmed of visible connective and adipose tissues.

The pH was measured using an Elmetron CP-411 pH meter with a dagger electrode calibrated at pH values of 4.0, 7.0 and 9.0.

The color of fresh and aged LL and GM samples was measured on the meat surface, after 30 min of exposure to the air, using a Minolta CR-410 (Konica-Minolta) colorimeter. The following color coordinates were determined: lightness (L${}^{*}$), redness (a${}^{*}$) and yellowness (b${}^{*}$), color saturation (C${}^{*}$) and hue (H${}^{*}$).

The LL and GM muscle samples were cut perpendicular to the muscle fiber into two parts and utilized for further analysis of expressed juice, cooking loss, shear force, chemical and fatty acid composition, as well as lipid peroxidation.

The expressed juice was determined by the Grau and Hamm (1953) method. Samples (0.3 g) of meat were placed on Whatman filter paper no. 1 and held under a pressure of 2 kg for 5 min. The outline area of the expressible juice and the meat film was traced, and two areas were measured using planimeter. The results have been calculated in cm${}^{2}$ g${}^{-1}$ meat.

To evaluate cooking loss, the samples of fresh and aged LL and GM muscles were weighed, wrapped in baking paper and heated in a convection oven at 90 ${}^{\circ }$C until they reached endpoint temperature (70 ${}^{\circ }$C) in the approximate geometric center of the sample. After cooking, the samples were cooled (40 min at 20–23 ${}^{\circ }$C), manually wiped with a paper towel to remove visible exudates and re-weighed to determine cooking losses.

The cooked samples were kept overnight in a chiller at 4 ${}^{\circ }$C. Then, the three cores (1.25 cm in diameter) were excised from each sample parallel to the muscle fiber orientation, through the thickest portion of the cooked muscle. Shear force was determined as maximum force (N) perpendicular to the fibers, using ZWIKI Roell type Z 2.5 equipped with a Warner–Bratzler blade. The average value for the sample was calculated based on three replications.

### 2.2 Chemical composition of the meat

The basic chemical composition of fresh and aged meat was determined by analyzing the contents of moisture, crude protein, intramuscular fat and collagen using a spectrometric technique with a near-infrared transmission (NIR) method (PN-A-82109). The meat samples were homogenized in an Elektrolux DITO K35 processor. Afterwards, unified samples were placed in a measuring cell of FoodScan analyzer. The device uses the near-infrared transmission method within 850–1050 nm range and is fitted with ANN calibration developed using a model of artificial neural networks. The analysis is performed by indicating in the computer program the number of 16 measurements in the sample, and then the program automatically calculates the average and presents the result.

### 2.3 Fatty acid analysis

The lipids from the fresh and aged muscle samples were extracted according to Folch et al. (1957). Saponification of fat was made in 0.5 M potassium hydroxide (KOH) in methanol and esterification in 10 % BF${}_{3}$ in methanol. The fatty acid methyl esters were extracted in the hexane.

The fatty acid profile of lipids was performed by gas-chromatograph analysis using the Agilent Technologies GC 6890 N instrument equipped with capillary column BP × 70 (length 60 m, internal diameter 0.22 mm, film thickness 0.25 µm). Operation conditions were helium gas (41 psi) and a FID detector at 240 ${}^{\circ }$C. The temperature program was 3 min at 130 ${}^{\circ }$C, an increase to 235 ${}^{\circ }$C by +2 ${}^{\circ }$C min${}^{-1}$; 4 min at 235 ${}^{\circ }$C.

The fatty acids were identified via reference material BCR 163 (beef/pig fat blend). The isomer linoleic acid (CLA) was determined by standard *cis*-9, *trans*-11 octadecadienoic acid-Larodon AB, Sweden.

### 2.4 Peroxidation of lipids

Lipid peroxidation products were evaluated in extracts from examined meat samples as thiobarbituric acid reactive substances (TBARS), according to the method of Uchiyama and Mihara (1978). Extracts were obtained after homogenization of tissue lyophilisates in radio-immunoprecipitation assay (RIPA) buffer and centrifugation (1600 × g, 10 min). TBARS were expressed as malondialdehyde (MDA) equivalents, and the precursor of MDA 1.2.3.3-tetraethoxypropane (TEP) was utilized as a standard. The absorbance was measured at 532 nm, applying a microplate reader (Infinite M200, Tecan, Männedorf, Switzerland).

### 2.5 Statistical analysis

A statistical analysis of the data obtained was performed using the SPSS 23.0 packet software (2016), based on a linear model that included the effect of muscle type and aging time. All effects were tested against residual middle squares to determine the level of significance. Tukey's test was used for comparing mean values when an F test for main effect was significant. The results are presented as the least squares means (LSMs) for each trait and standard error (SE).

**Table 2:** The mean values for meat quality properties for different muscles and aging time.

Item	Fresh meat		Meat aged 14 d		SE
	LL	GM	LL	GM	
pH	5.56	5.55${}^{a}$	5.61	5.69${}^{b}$	0.03
L${}^{*}$ (lightness)	39.67	40.73${}^{a}$	39.92${}^{x}$	36.73${}^{\mathrm{by}}$	0.42
a${}^{*}$ (redness)	15.26	16.25${}^{a}$	15.85${}^{x}$	17.34${}^{\mathrm{by}}$	0.30
b${}^{*}$ (yellowness)	5.71	5.88${}^{a}$	5.93${}^{x}$	7.06${}^{\mathrm{by}}$	0.29
C${}^{*}$	16.35	17.42${}^{a}$	17.04${}^{x}$	18.84${}^{\mathrm{by}}$	0.30
H${}^{*}$	20.45	19.99	20.36	22.29	0.97
Expressed juice (cm${}^{2}$ g${}^{-1}$)	20.50${}^{a}$	19.65${}^{a}$	14.73${}^{b}$	12.60${}^{b}$	0.80
Cooking loss (%)	28.91${}^{\mathrm{ax}}$	37.25${}^{y}$	34.48${}^{b}$	34.64	0.97
Shear force (N)	59.43${}^{\mathrm{ax}}$	40.86${}^{\mathrm{ay}}$	43.67${}^{\mathrm{bx}}$	35.45${}^{\mathrm{by}}$	1.61

LL – *M. longissimus lumborum*. GM – *M. gluteus medius*. SE – standard error. ${}^{a}$, ${}^{b}$ – different superscripts in the same row represent significant differences among aging time (within individual muscles) (P<0.05). ${}^{x}$, ${}^{y}$ – different superscripts in the same row represent significant differences among individual muscles (within aging time) (P<0.05).

## 3 Result and discussion

### 3.1 Physical characteristics of meat

There were no statistical differences in the pH value between analyzed LL and GM muscles both in fresh and aged meat. The pH value increased slightly after 14 d of aging but the statistically significant differences (P<0.05) were recorded only for GM muscle (Table 2). In the studies of Abdullah and Qudsieh (2009), aging for 7 d had no effect on pH in *semitendinosus*, *semimembranosus*, *biceps femoris* and *longissimus* muscles. In turn, Yanar and Yetim (2001) reported that pH in *longissimus dorsi* and *semimembranosus* muscles from adult ewes significantly declined after 7 d of the aging period. The pH value in the examined LL and GM muscles in both aging periods (24 h and 14 d) was lower than that obtained by Tschirhart-Hoelscher et al. (2006) in *longissimus* and *gluteus medius* muscles after 7 d of aging.

The aging time has an effect on the changes in meat color. The increase (P<0.05) of the value of redness (a${}^{*}$) and yellowness (b${}^{*}$) and decline (P<0.05) of lightness (L${}^{*}$) was recorded in the GM muscle after the 14 d aging period (Table 2). The greater saturation of redness was confirmed by the increase of C${}^{*}$ value (P<0.05) in *gluteus medius* muscle. The aging had no effect on lightness, redness and yellowness, as well as chroma (C) and hue angle (H) in relation to the *longissimus lumborum* muscle (Table 2). Differences in the parameters characterizing the color of meat between the examined LL and GM muscles were not found in fresh meat, but the value of these parameters among both muscles differs significantly after 14 d of aging. The LL muscle was characterized by higher values of L${}^{*}$ and lower redness, yellowness and chroma in comparison with the GM muscle (Table 2). The presented results are different than those reported by Tschirhart-Hoelscher et al. (2006), who, analyzing the color of 18 lamb skeletal muscles after a 7 d aging period, recorded a higher lightness value of the *gluteus medius* muscle (L${}^{*}\;=43.2$) and *longissimus lumborum* (L${}^{*}=42.7$), lower redness and yellowness, which for GM and LL muscle were a${}^{*}$ – 16.5 and 14.7, b${}^{*}$ – 4.3 and 3.8, respectively. In turn, Abdullach and Qudsieh (2009), in different muscles of Awassi lambs compared to fresh meat, noted a significant increase in a${}^{*}$ values with a slight decrease in L${}^{*}$ after 7 d of aging. According to McKenna et al. (2005), changes in L${}^{*}$ values during the aging process are minor and play a minimal role in color stability of red meat. The influence of storage time on changes in the color characteristics of lamb meat was confirmed by Luciano et al. (2012) and Ripoll et al. (2013), pointing to the complexity of conditions deteriorating the acceptability of the color of meat by consumers.

The meat color is determined primarily by myoglobin, the concentration of which in skeletal muscles may depend on their physiological activity and the muscle fiber type in their structure (Mancini and Hunt, 2005). The myoglobin concentration is higher in muscles containing more slow oxidative than fast glycolytic fibers (Picard et al., 2002). This may explain the differences in the parameters determining the color between LL and GM muscles obtained in the conducted studies. According to Ithurralde et al. (2015), the proportion of slow oxidative fibers in the *gluteus medius* muscle is greater than in the *longissimus lumborum*. The greater differences between examined muscles observed in the aged meat may be a consequence of the transformation of myoglobin into other forms responsible for the less desirable meat color.

The aging time significantly affected (P<0.05) the cooking loss in respect to the LL muscle. The differences in the cooking loss between fresh meat and meat aged for 14 d in the GM muscle were not recorded; however, the value of this parameter measured on fresh meat (24 h after slaughter) was higher (P<0.05) in the *gluteus medius* muscle than in the *longissimus lumborum* (Table 2). As in the present study, the increase in cooking loss in *longissimus lumborum* muscle after 5 d of aging was recorded by Vieira and Fernandez (2014) in suckling lambs. The increase of this parameter in the lamb *semimembranosus* muscle after 7 d of aging was also reported by Florek et al. (2018). In contrast, in the research of Abdullah and Qudsieh (2009), aging caused a significant decrease in this parameter in Awassi lambs' *longissimus* muscle. The aging time did not significantly affect the cooking loss in other muscles examined by the abovementioned authors.

Analyzing the expressed juice, it was found that the meat after the 14 d aging period was characterized by a better value of this parameter. The lower value (P<0.05) of this characteristic was found in both LL and GM muscles. The similar results were given by Abdullah and Qudsieh (2009) for *semitendinosus*, *semimembranosus*, *biceps femoris* and *longissimus* lamb muscles. The lower ability of water holding in fresh meat may be caused by the increase in the space between myofilaments due to the sarcomere shortening *rigor mortis* process. During meat aging, the proteolytic degradation of cytoskeletal proteins results in the loosening of the sarcomere structure and may allow the meat to retain water (Pearce et al., 2011).

In the present study, the meat tenderness, which is one of the most important features in consumer assessment, was better in aged meat. Both LL and GM muscles that were aged for 14 d had significantly (P<0.05) lower shear force values than those in fresh meat (Table 2). The effect of the time of meat aging on increasing its tenderness was noted by Florek et al. (2018) in lamb and veal meat, Abdullah and Qudsieh (2009) in different muscles of Awassi ram lambs and Sosin-Bzducha and Puchała (2017) in beef. The better tenderness of aged meat could be associated with changes in myofibrillar structure predominantly due to proteolysis of cytoskeletal proteins, degradation of Z disks and some regulatory proteins (Koohmaraie, 1996). These processes cause a weakening of myofibrils and muscle relaxation contributing to the improvement of tenderness. The key role in meat tenderization is played by calpains, proteins belonging to the family of calcium-dependent cysteine proteases (Koohmaraie and Geesink, 2006).

The shear force in the present study was affected by muscle type (Table 2). The shear force value was higher (P<0.05) in *longissimus lumborum* compared to *gluteus medius* muscle in fresh and 14 d aged meat. Differences in the shear force in the same muscles as in the presented studies in fresh and 7 d aged meat were noted by Ablikim et al. (2016) and Tschirhart-Hoelscher et al. (2006). However, the results obtained by these authors indicated a greater value of shear force in the *gluteus medius* muscle than in the *longissimus lumborum*. The differences in the shear force depending on the type of muscles were recorded in lamb meat also by Esenbuga et al. (2009) and by Kopuzlu et al. (2018) in beef. According to Abdullah et al. (2011), the variation in shear force values between muscles may result from differences in sarcomere length, muscle fiber types and their diameters.

### 3.2 Chemical composition of meat

The results of chemical composition of fresh and aged meat are given in Table 3. Compared to fresh meat, the moisture content in both LL and GM muscles was lower (P<0.05) after 14 d of aging. The moisture in the fresh LL muscle was higher (P<0.05) in comparison to that in the GM muscle. The influence of the storage time of meat on its water content has also been confirmed by Ablikim et al. (2016). However, in the research of Abdullah and Qudsieh (2009), the moisture content was not affected by aging time in all investigated muscles of Awassi lambs.

**Table 3:** The mean values for meat chemical composition for different muscles and aging time.

Item	Fresh meat		Meat aged 14 d		SE
	LL	GM	LL	GM	
Moisture (%)	74.35${}^{\mathrm{ax}}$	73.76${}^{\mathrm{ay}}$	73.09${}^{b}$	73.08${}^{b}$	0.21
Protein (%)	20.29	22.79	21.34	22.29	0.13
Fat (%)	4.77${}^{x}$	3.78${}^{y}$	5.39${}^{x}$	4.07${}^{y}$	0.27
Total collagen (%)	1.13${}^{\mathrm{ax}}$	1.84${}^{\mathrm{ay}}$	1.73${}^{\mathrm{bx}}$	2.02${}^{\mathrm{by}}$	0.05

LL – *M. longissimus lumborum*. GM – *M. gluteus medius*. SE – standard error. ${}^{a}$, ${}^{b}$ – different superscripts in the same row represent significant differences among aging time (within individual muscles) (P<0.05). ${}^{x}$, ${}^{y}$ – different superscripts in the same row represent significant differences among individual muscles (within aging time) (P<0.05).

Statistically significant differences in the moisture content between different lamb muscles were also noted by Esenbuga et al. (2009), whereas Ablikim et al. (2016) did not find differences in this parameter among *longissimus dorsi* and *gluteus medius* muscles in fresh meat.

The aging time had no effect on the protein and fat content in the examined muscles (Table 3). Nevertheless, both parameters reached higher values in the LL and GM muscles after 14 d of aging. The obtained results are in agreement with those presented by Abdullah and Qudsieh (2009), who found that the crude protein and fat content were not influenced by meat aging. In the present study, the fresh and aged LL muscles were characterized by the higher (P<0.05) intramuscular fat content compared to the GM muscle. In turn, Ablikim et al. (2016) did not report differences in fat content between *longissimus dorsi* and *gluteus medius* muscles, and neither did Esenbuga et al. (2009) when comparing the *longissimus dorsi*, *semitendinosus* and *triceps brachii* muscles.

**Table 4:** The mean values for fatty acid composition and TBARS for different muscles and aging time.

Item	Fresh meat		Meat aged 14 d		SE
	LL	GM	LL	GM	
TBARS (mmol g${}^{-1}$ meat)	0.35${}^{x}$	0.58${}^{y}$	0.42${}^{x}$	0.65${}^{y}$	0.03
C10:0	0.10	0.08	0.11	0.10	0.01
C12:0	0.10	0.07	0.08	0.07	0.01
C14:0	1.96${}^{x}$	1.76${}^{y}$	2.08${}^{x}$	1.79${}^{y}$	0.07
C15:0	0.35${}^{a}$	0.37	0.39${}^{b}$	0.38	0.01
C16:0	24.29${}^{x}$	22.80${}^{y}$	24.06${}^{x}$	22.92${}^{y}$	0.32
C17:0	1.51	1.62	1.60	1.63	0.06
C18:0	17.52	17.71	17.56	17.63	0.41
C20:0	0.11	0.11	0.10	0.10	0.01
C14:1	0.05${}^{x}$	0.04${}^{y}$	0.06${}^{x}$	0.05${}^{y}$	0.00
C16:1	2.08	2.01	2.11	2.05	0.04
C17:1	0.78	0.81	0.84	0.81	0.04
Σ C18:1t	4.92	4.86	5.29	4.84	0.26
C18:1 n9	38.06	38.51	37.88	38.27	0.36
C18:1 n7	1.19	1.23	1.14	1.23	0.03
C20:1	0.15	0.13	0.15${}^{x}$	0.12${}^{y}$	0.01
Σ C18:2 t	0.12	0.12	0.12	0.12	0.01
C18:2 n6	3.30${}^{x}$	3.86${}^{y}$	3.33${}^{x}$	3.94${}^{y}$	0.18
C20:2	0.06	0.06	0.06	0.07	0.01
C18:3 n3	1.08	1.10	1.07	1.06	0.02
C20:3 n6	0.08${}^{x}$	0.11${}^{y}$	0.07${}^{x}$	0.10${}^{y}$	0.01
C20:4 n6	0.68${}^{x}$	1.01${}^{y}$	0.60${}^{x}$	0.96${}^{y}$	0.06
C20:5 n3	0.07${}^{x}$	0.10${}^{y}$	0.08${}^{x}$	0.10${}^{y}$	0.01
C22:5 n3	0.18${}^{x}$	0.23${}^{y}$	0.17	0.21	0.01
C22:6 n3	0.05	0.06	0.06	0.06	0.01
C18:2c9t11(CLA)	0.39${}^{x}$	0.33${}^{\mathrm{ay}}$	0.37	0.37${}^{b}$	0.01
Σ SFA	45.94${}^{x}$	44.51${}^{y}$	45.98${}^{x}$	44.62${}^{y}$	0.44
Σ MUFA	47.23	47.60	47.47	47.37	0.28
Σ PUFA	6.00${}^{x}$	6.97${}^{y}$	5.94${}^{x}$	6.97${}^{y}$	0.25
Σ n6	4.05${}^{x}$	4.97${}^{y}$	4.01${}^{x}$	4.99${}^{y}$	0.23
Σ n3	1.38	1.49	1.38	1.42	0.04
n6 / n3	2.97${}^{x}$	3.36${}^{y}$	2.91${}^{x}$	3.50${}^{y}$	0.14

LL – *M. longissimus lumborum*. GM – *M. gluteus medius*. SE – standard error. ${}^{a}$, ${}^{b}$ – different superscripts in the same row represent significant differences among aging time (within individual muscles) (P<0.05). ${}^{x}$, ${}^{y}$ – different superscripts in the same row represent significant differences among individual muscles (within aging time) (P<0.05).

Meat aging resulted in an increase (P<0.05) of total collagen content in both LL and GM muscles. In addition, the significant differences were found between investigated muscles. The content of total collagen was higher (P<0.05) in *gluteus medius* compared to *longissimus dorsi* in fresh and aged meat (Table 3). The effect of aging on total collagen content has not been confirmed by Kołczak et al. (2008) in bovine *semitendinosus* and *psoas major* muscles. In turn, Geldenhuys et al. (2016) found a significant increase in total collagen in the *pectoralis major* muscle in geese after 14 d of aging compared to not aged meat. The differences in the total collagen content depending on the type of muscle in lamb meat were noted by Tschirhart-Hoelscher et al. (2006). As in the present study, authors found the higher total collagen content in *gluteus medius* muscle compared to *longissimus lumborum*, whereas Ablikim et al. (2016) noted a higher content of collagen in the *longissimus dorsi* muscle than in the *gluteus medius*. The differences in total collagen content between different muscles may be related to the intramuscular fat content. According to Listrat et al. (2016), a negative correlation between collagen content and intramuscular fat has been observed. It indicates that an increase in fat would cause a relative decrease in muscle collagen content, which was also confirmed in the present study (Table 3).

### 3.3 Lipid oxidation and fatty acid profile

In the conducted study, the aging time increased the rate of lipid peroxidation expressed as TBARS; however, the differences were not statistically confirmed (Table 4). The higher content of substances reacting with thiobarbituric acid (P<0.05) was recorded in the GM muscle compared to *longissimus lumborum* both in fresh and 14 d aged meat (Table 4). The increase of lipid oxidation in the meat of crossbred lambs was obtained by Ponnampalam et al. (2017) but as a result of 60 d of aging. In the studies of Ripoll et al. (2013), the oxidation rate increased after 7 d of aging LL muscle of Aragonesa ram lambs, although it was different depending on the ration composition and the level of α-tocopherol supplementation. The increase in peroxidation process under the influence of aging time depending on type of nutrition was recorded by Luciano et al. (2012).

The oxidative stability of meat depends primarily on balance between the antioxidant and pro-oxidative components in the meat (Descalzo and Sancho, 2008). The polyunsaturated fatty acids are substances undoubtedly initiating lipid oxidation. On the other hand, both endogenous and exogenous defense mechanisms that counteract oxidation are able to extend the shelf life, which may explain insignificant oxidative changes in aged meat in the present study.

In turn, the differences in TBARS values between investigated muscle types may be a consequence of significant differences in PUFA content. The higher amount of this acid group (P<0.05) was found in fresh and aged GM muscle compared to LL (Table 4). In the *gluteus medius* muscle, the proportion of long-chain acids with more than two numbers of unsaturated bonds, which are less resistant to oxidation (C20:3; C20:4; C20:5), was higher (P<0.05) in both investigated periods. As reported by Yang et al. (2002), even small changes in the concentration of such acids can have a significant effect in oxidative stability of meat. On the other hand, the increased content of PUFAs may enhance the antioxidant activity and counteract these processes. Similarly, being a good antioxidant itself, CLA isomer (C18:2 *cis*-9, *trans*-11), can provide natural protection against lipid oxidation (Decker, 1995). Increased content (P<0.05) of this isomer in the GM muscle after 14 d of aging could have a positive effect on oxidative stability (Table 4). In addition to the abovementioned isomer, the meat aging had no effect on the fatty acid content in both LL and GM muscles. Similarly, Mungure et al. (2016) did not register changes in SFA, MUFA and PUFA content in heifers' *semimembranosus* muscle between 7 and 14 d of aging.

The results of other authors (Carnevale de Almeida et al., 2006; Mourot et al., 2015; De Brito et al., 2017) suggest that the fatty acid profile, apart from many factors, may be affected by muscle type, regardless of the animal species. The higher content (P<0.05) of n-6 PUFA in the GM muscle in the present study confirmed the results of Janovska et al. (2010), who indicated that muscles with a higher proportion of oxidative fibers have a greater ability to accumulate this family of acids. A higher proportion of n-6 acids resulted in an increase (P<0.05) of the n-6 / n-3 ratio in the *gluteus medius* muscle.

## 4 Conclusions

The results of this study indicate that the aging process had an effect on some physicochemical characteristics of lamb meat. Meat subjected to 14 d of aging was characterized by a darker color, larger cooking loss and the better parameters of expressed juice. Aged meat, despite higher collagen content, showed significantly better tenderness, which is very important in terms of consumer acceptance. The aging time had no effect on the oxidative stability and fatty acid profile.

The differences between LL and GM muscles concerned the meat color, shear force value, fatty acid profile and lipid oxidation. The LL muscle was lighter in color, while the GM muscle had better tenderness. The higher PUFA content in the GM muscle, especially from the n-6 family, resulted in an increased n-6 / n-3 ratio. The TBARS value indicated better oxidative stability of the LL muscle.

## Data Availability

The data are available from the corresponding author upon request.
